# Transmission dynamics, clinical characteristics and sero-surveillance in the COVID-19 outbreak in a population dense area of Colombo, Sri Lanka April- May 2020

**DOI:** 10.1371/journal.pone.0257548

**Published:** 2021-11-08

**Authors:** Chandima Jeewandara, Dinuka Guruge, Deshni Jayathilaka, Panambara Arachchige Deshan Madhusanka, Pradeep Darshana Pushpakumara, Shyrar Tanussiya Ramu, Inoka Sepali Aberathna, Danasekara Rallage Saubhagya Rasikangani Danasekara, Thilagaraj Pathmanathan, Buddhini Gunatilaka, Sauni Malavige, Yowan Dias, Ruwan Wijayamuni, Graham S. Ogg, Gathsaurie Neelika Malavige

**Affiliations:** 1 Allergy Immunology and Cell Biology Unit, Department of Immunology and Molecular Medicine, University of Sri Jayewardenepura, Nugegoda, Sri Lanka; 2 Colombo Municipal Council, Colombo, Sri Lanka; 3 MRC Human Immunology Unit, MRC Weatherall Institute of Molecular Medicine, University of Oxford, Oxford, United Kingdom; University of Zambia, ZAMBIA

## Abstract

**Background:**

The transmission dynamics of SARS-CoV-2 varies depending on social distancing measures, circulating SARS-CoV-2 variants, host factors and other environmental factors. We sought to investigate the clinical and epidemiological characteristics of a SARS-CoV-2 outbreak that occurred in a highly dense population area in Colombo, Sri Lanka from April to May 2020.

**Methodology/principal findings:**

We carried out RT-qPCR for SARS-CoV2, assessed the SARS-CoV-2 specific total and neutralizing antibodies (Nabs) in a densely packed, underserved settlement (n = 2722) after identification of the index case on 15^th^ April 2020. 89/2722 individuals were detected as infected by RT-qPCR with a secondary attack rate among close contacts being 0.077 (95% CI 0.063–0.095). Another 30 asymptomatic individuals were found to have had COVID-19 based on the presence of SARS-CoV-2 specific antibodies. However, only 61.5% of those who were initially seropositive for SARS-CoV-2 had detectable total antibodies at 120 to 160 days, while only 40.6% had detectable Nabs. 74/89 (83.1%) of RT-qPCR positive individuals were completely asymptomatic and all 15 (16.9%) who experienced symptoms were classified as having a mild illness. 18 (20.2%) were between the ages of 61 to 80. 11/89 (12.4%) had diabetes, 8/89 (9%) had cardiovascular disease and 4 (4.5%) had asthma. Of the two viruses that were sequenced and were of the B.1 and B.4 lineages with one carrying the D614G mutation.

**Discussion/conclusion:**

Almost all infected individuals developed mild or asymptomatic illness despite the presence of comorbid illnesses. Since the majority of those who were in this underserved settlement were not infected despite circulation of the D614G variant, it would be important to further study environmental and host factors that lead to disease severity and transmission.

## Introduction

SARS-CoV-2 has currently affected 221 countries and is the leading cause of mortality in USA, some Latin American countries and in certain regions in Europe [1]. The World Health Organization estimated that 10% of the global population may have already been infected with the virus by October 2020 [2]. The transmission dynamics of the virus varies depending on social distancing measures, circulating SARS-CoV-2 virus variants, host factors and other environmental factors [3–5]. The extent of infection, the route of transmission, the full range of disease presentation and the viral dynamics may vary based on the setting. Understanding the epidemiological, clinical and characteristics of the virus in the selected communities that were affected with COVID-19 and their close contacts is imperative to inform targeted measures for the public health response.

The first patient infected with SARS-CoV-2 in Sri Lanka was reported on the 27^th^ January, who was a foreign national and the first Sri Lankan patient was reported on the 10^th^ of March [6]. Since then, the spread of the virus was largely contained until end of September 2020, and only 3111 cases were reported within a period of 6 months (March to September, 2020), of which 38.8% were imported cases (acquired the infection outside Sri Lanka) [7]. Among many public health measures, extensive restriction of movements of people through ‘lockdowns’ was one main measure used by the government to control the situation. However, in early April, during the island wide lockdown period, limited spread was picked up during community screening in a population dense, underserved settlement withing the Colombo metropolitan region. This outbreak was detected in Bandaranayaka *watta* within the legislative premises of Colombo Municipal Council (CMC) in the City of Colombo.

**Fig 1:**
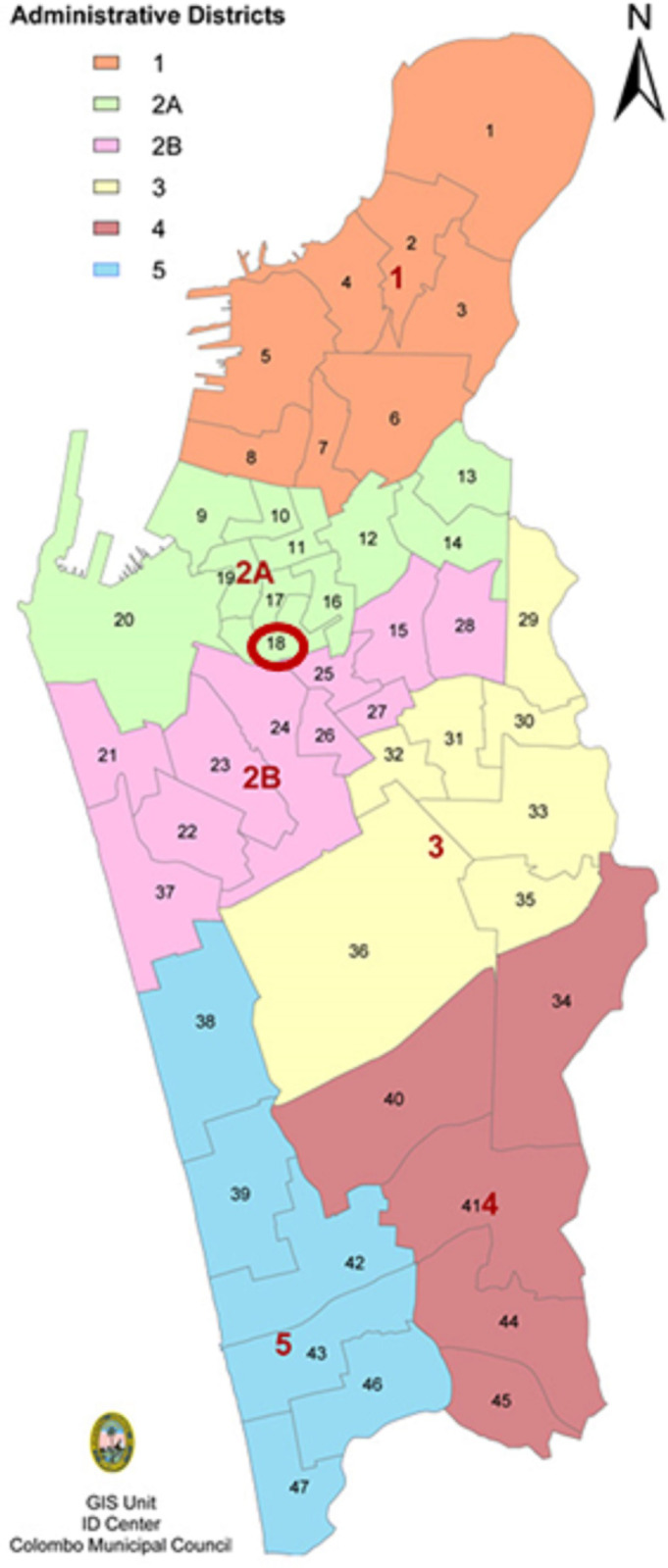
A map of the District 2A areas of the Colombo municipality council. The location of the Bandaranayaka watta is circled in red. The figure is from GIS unit ID Center, Colombo Municipal Council Sri Lanka.

The City of Colombo, the commercial capital of the country is spread across an area of 37.3 km² and has a 750000 resident population and 0.5 million population of daily migrants. It’s the most populated city in Sri Lanka. Colombo City is divided into five administrative divisions (1, 2A, 2B, C, D, E) and Bandaranayaka *watta* is located in the 2A Division (Fig 1). It is an underserved settlement of approximately 6,955 square meters (0.007 km^2^) with 2722 persons in 464 families living in slum houses, which are much overcrowded (population density; 388,857.1 inhabitants/km^2^)

The index case of the outbreak of COVID-19 detected on 15 April 2020 in the Bandaranayaka *Watta* was a symptomatic 59-year-old female. She was a returnee from a pilgrimage to India and after discharge from a 14-day mandatory quarantine following a negative RT-qPCR, she presented to hospital with chest pain. The RT-qPCR done on admission gave a positive result, which was after 45-days from her return to Sri Lanka.

An epidemiological investigation for further cases and contacts using a risk stratified approach was initiated immediately in the Bandaranayaka *watta* by the Public Health Department of CMC and the Epidemiological Unit of the Ministry of Health. The methods for sero-surveillance was based on the Unity studies of WHO [8]. In order to understand the transmission dynamics within a very highly populated area, we proceeded to study the epidemiological and the virus characteristics and sero-survillence in individuals living in this region.

## Materials and methods

### Study participants

This study was conducted prospectively for a period of 65 days following the detection of the index case on 15^th^ April 2020 (15 April 2020 to 19 May 2020). Nasopharangeal swabs were obtained by field health staff from all study participants (all close contacts and all non-close contacts to determine if they were infected with the SARS-CoV-2). The first 3 cases were detected within the 24 hours of detection of index case, and therefore, they were also classified as “primary cases”.

Close contacts were defined as individuals living in Bandaranayaka *watta* and who had direct physical contact or associated with cases (distance of 1m) within a period of 2 days from identification of the index case. Therefore, individuals who had contact with symptomatic, index cases 2 days prior to their diagnosis were traced back. Since this outbreak was during a ‘locked-down’ period with enforcement of a police curfew, residents of “Bandaranayaka watta” were confined to their homes. All RT-qPCR positive, close contacts were classified as cases and were hospitalized. RT-qPCR negative contacts were directed to a quarantine facility for 14 days to ensure that they stay isolated under observation of health staff. They were retested at the end of the quarantine period, irrespective of presence of absence of symptoms.

The ‘non-close contacts’ were defined as those who were living within the CMC region as the cases, or those who worked with cases in the same causal occupations but were not qualified to be defined as close contacts. The investigations by the health staff for each of the cases of Bandaranayaka watta resulted in identification of the ‘non-close contacts’. The RT-qPCR positive ‘non-close contacts’ were classified as cases and were directed them to hospitals. If RT-qPCR was negative, they were directed to isolate themselves in their own homes for 14 days, under the observation of health staff according to government regulations at the time. They were retested after the quarantine period, irrespective of presence or absence of symptoms. In case if they develop symptoms after the quarantine period, again they were retested. The methods for sero-surveillance was based on the Unity studies of WHO [8].

### Data collection

Demographic and clinical data were collected from all the study participants (cases, close contacts and non-close contacts) following informed written consent. Quarantine center records and field health records were used as source of data. Clinical disease severity was classified as mild, moderate and severe according to the WHO guidance of COVID-19 disease severity [9].

### Ethics statement

Ethics approval was obtained by the Ethics Review Committee of University of Sri Jayewardenepura (COVID 01/20). All participants gave informed written consent and for child participants, the informed written consent of the parents was obtained.

### Sample collection

Two blood samples were obtained from all cases (individuals who were infected with the SARS-CoV-2) and close contacts who were recruited for the study while only one blood sample was obtained from non-close contacts who were recruited to the study. The first sample was obtained from cases (n = 89) close contacts (n = 452) and non-close contacts (n = 1539) between day 11 to 60 day from identification of the index patient. The second blood sample was obtained 120 to 160 days from the identification of the index patient in those who had COVID-19 (n = 66) and their close contacts (n = 439). During the second blood sample collection, only a subset of participants volunteered to provide a blood sample. A second blood sample was not obtained from non-close contacts due to logistics.

### RT-qPCR for detection of SARS CoV-2

Nasopharyngeal swabs of suspected SARS- CoV-2 patients were lysed and RNA was extracted using QIAmp Viral RNA Mini Kit (Qiagen, USA, Cat: 52906) and used to detect the presence of N gene and ORF1ab gene of SARS-CoV2 with Da An Gene real time PCR kit (Da An Gene, China. Cat: DA-930) by RT-qPCR according to manufacturers’ instructions in ABI 7500 real time PCR system (Applied Biosystems, USA).

### Assay to measure total antibodies and neutralizing antibodies (Nabs)

Due to the limitations in using a BSL-3 facility to carry out assays to measure neutralizing antibodies, the Nabs were measured using a surrogate virus neutralization test (sVNT) [10]. This is an indirect measure of the presence of Nabs as it measures the percentage of inhibition of binding of the RBD of the S protein to recombinant ACE2 (Genscript Biotech, USA). Inhibition percentage ≥ 25% in a sample was considered as positive for Nabs. We have validated this in the Sri Lanka population and in patients with SARS-CoV2 previously [11].

SARS-COV-2 Total antibody responses were assessed using WANTAI SARS-CoV-2 Ab ELISA (Beijing Wantai Biological Pharmacy Enterprise, China). The assay was carried out and results were interpreted according to manufactures instructions. The specificity of the assay was assessed using 81 serum samples obtained from National Institute of Infectious Diseases, Sri Lanka in 2018, and all gave a negative result. All samples had absorbance less than the cut off value for the assay. Therefore, the assay had 100% specificity for Sri Lankan individuals.

Using these assays, we measured total antibodies and Nabs (by sVNT) in all blood samples of cases (n = 89) and close contacts (n = 437) during the first 11–60 days since detection of index case. Assays for total antibodies were also done in the 1539 non-close contacts. However, in the non-close contacts, Nabs were measured in only 14/23 who gave a positive result with the total antibody assay, due to limited sample volume. In samples collected during day 120–160 from the initial detection of the index case, total antibodies were measured in 65 cases and 418 close contacts while Nabs were measure in 64 cases and 414 close contacts.

### Library preparation and Next Generation Sequencing (NGS) and phylogenetic analysis of SARS-CoV-2 sequences

We carried out whole genomic sequencing of two of the viruses from these 89 patients, who had Ct values of <25. Library preparation was attempted using the AmpliSeq for Illumina SARS-CoV-2 Community Panel, in combination with AmpliSeq for Illumina library prep, index, and accessories (Illumina, San Diego, USA) and targeted RNA/cDNA amplicon assay was used. The representative lineage sequences were downloaded from https://github.com/cov-lineages/lineages (anonymised.encrypted.aln.safe.fasta) on 16th September 2020. GISAID (https://raw.githubusercontent.com/hCoV2019/lineages/master/gisaid_acknowledgements.tsv) [12]. Sequence lineage, nucleotide mutations and amino acid replacements were generated using the CoV-GLUE graphical user interface [13].

## Results

Following the identification of the index case, three immediate contacts were identified to have been infected with COVID-19 by RT-qPCR and an epidemiological investigation identified 1093 individuals as close contacts, and 1625 individuals as non-close contacts. 85 individuals among close contacts were confirmed as infected while none were identified among the non-close contacts. The age and demographic characteristics of these individuals are shown in Table 1. 39.3% of those who were infected were between the ages of 31 to 60 years. Only 1 individual was older than 80 years. The highest number of cases were identified on 20 April 2020, 5 days after the detection of the primary case and 87 of the 89 cases were detected by 26 April, 11 days after the onset.

**Table 1:** Demographic characteristics of the infected individuals and their contact.

Characteristic	Cases	Close contacts	Non-close contacts
	N = 89	N = 1008	N = 1625
**Sex**			
Males (%)	47 (52.8)	694 (68.8)	1356 (83.4)
Females (%)	42 (47.2)	314 (31.2)	269 (16.6)
**Age category**			
<5 years (%)	4 (4.5)	28 (2.8)	5 (0.3)
6–15 years (%)	11 (12.4)	79 (7.8)	15 (0.9)
16–30 years (%)	20 (22.5)	269 (26.7)	336 (20.7)
31–60 years (%)	35 (39.3)	528 (52.4)	1120 (68.9)
>60 years	19 (21.3)	104 (10.3)	149 (9.2)
**Ethnicity**			
Sinhalese (%)	8 (9.0)	303 (30.1)	1088 (67.0)
Tamil (%)	7 (7.9)	145 (14.4)	292 (18.0)
Muslim (%)	74 (83.1)	532 (52.8)	187 (11.5)
Burgher (%)	0 (0)	28 (2.8)	58 (3.6)

As 85/1093 individuals who were identified as close contacts of the initial four COVID-19 patients, subsequently became positive, the secondary attack rate among close contacts was 0.08 (95% CI 0.06–0.09) with secondary clinical attack rate being 0.01 (95% CI 0.001–0.02). The 95% confidence intervals for the secondary clinical attack rates were calculated as previously described [14]. The secondary attack rate was defined as frequency of new COVID-19 infection among contacts of a confirmed case in a defined period of time. In the calculation of secondary attack rate, the first 3 cases detected within the 24 hours along with the index case were classified as “primary cases”. The secondary clinical attack rate was defined as the frequency of new symptomatic cases of COVID-19 infection among the contacts of confirmed cases in a defined period of time, as determined by a positive RT-qPCR result. Infection was not detected by RT-qPCR in any of the non-close contacts.

### Clinical disease severity of COVID-19 in this cohort

Out of 89 RT-qPCR positive patients, 15 complained of symptoms at the time of diagnosis or prior to diagnosis. The commonest were sore throat (6/89, 6.7%), cough (5/89, 5.6%), runny nose (2/89, 2.2%) and fever (2/89, 2.2%). 74/89 (86.5%) were completely asymptomatic throughout the 14 days of mandatory hospitalization or quarantine period. All 15 (16.9%) who experienced symptoms were classified as having a mild illness based on the WHO classification of COVID-19 clinical disease severity [9]. 11/89 (12.4%) had diabetes, 8/89 (9%) had cardiovascular disease and 4 (4.5%) had asthma.

### Presence of SARS-CoV2 specific antibodies in the cohort

SARS-CoV-2 specific total antibodies were detected in 59/89 (67.8%%) individuals who were identified as having COVID-19, based on a positive RT-qPCR result, 5/452 (1.1%) of individuals identified as close contacts, also had detectable total antibodies, despite being RT-qPCR negative (Table 2). Of all non-close contacts were tested by RT-qPCR, 23/1539 (1.5%), who were also negative for RT-qPCR had SARS-CoV2 specific total antibodies. NAbs were detected in all 59 individuals who were having COVID-19 (67.8%). In the second sample obtained between 120 to 160 days since detection of the indexed patient, 40/65 (61.5%) SARS-CoV-2 infected individuals had detectable SARS-CoV-2 specific total antibodies, whereas only 26/64 (40.6%) had neutralizing antibodies. Of the close contacts of those who were found to be SARS-CoV-2 RT-qPCR positive, 7/439 (1.7%) had detectable SARS-CoV-2 specific total antibodies at the second time point, whereas only 1/439 (0.2%) had detectable Nabs.

**Table 2:** The presence of SARS-CoV-2 specific total antibodies and neutralizing antibodies in COVID-19 patients, their close contacts and non-close contacts in the Colombo Municipality region.

	SARS-CoV antibody positivity at 11 to 60 days		SARS-CoV antibody positivity at 120 to 160 days	
	Total antibodies	Neutralizing antibodies	Total antibodies	Neutralizing antibodies
COVID-19 RT-qPCR positive patients	59/89 (67.8%)	59/89 (67.8%)	40/65 (61.5%)	26/64 (40.6%)
Close contacts	5/437 (1.1%)	4/437 (0.9%)	7/418 (1.7%)	1/414 (0.2%)
Non-close contacts	23/1539 (1.5%)	0/14	NA	NA

During the second wave of COVID-19 that Sri Lanka is currently experiencing, many areas within the CMC including Bandaranayaka watta was affected. 11 individuals of this Bandarayanaka watta were found to be infected by RT-qPCR (previously uninfected individuals), while one previously infected individual also became re-infected. Although his RT-qPCR was positive for SARS-CoV-2 in April, he had not developed any detectable SARS-CoV2 specific total antibodies or Nabs.

### Virus characteristics

We sequenced two viruses (only 2/89 samples had RT-qPCR Ct values <25) from this cohort which revealed that they were of clades B.4 and B.1 (Table 3), suggesting that many different virus strains were circulating within the Bandaranayaka watta during this time. One of the viruses had the D614G mutation.

**Table 3:** Type and number of mutations and the Pangolin lineage details of the SARS-CoV-2 viruses sequenced during this outbreak.

Sample ID (GISAID ID)	Date of collection	Lineage	Number of SNPs	Nucleotide Mutations	Amino acid replacements
CDR142 (EPI_ISL_525474)	4/19/2020	B.4	16	G1397A,T3873C,C5986T,G11083T,A11506T,C11758T,C11986T,G18984C,C20483T,C21380T,G22770A,G25684T,T26181G,C27879T,T28688C,G29465T	ORF1a:V378I,
					ORF1a:L3606F,
					ORF1b:M1839I,
					ORF1b:S2339F,
					ORF3a:A98S,
					ORF3a:I263M,
					ORF7b:H42Y,
					N:A398S
CDR1885 (EPI_ISL_525476)	4/28/2020	B.1	14	C3037T,C3093T,G10523A,C14408T,C15324T,G16117A,A21550C,A21551T,A23403G,C27213T,A27379G,G27382C,A27383T,T27384C	ORF1a:P943L,
					ORF1a:V3420I,
					ORF1b:P314L,
					S:D614G,
					ORF6:I60V

## Discussion

Understanding the transmission dynamics of SARS-CoV-2 infections could provide critically important implications for the prevention and control of COVID-19 worldwide. In this study we have investigated the spread of the virus and detection rates by RT-qPCR compared to SARS-CoV-2 specific total and Nabs. The location of the outbreak, Bandaranayaka *watta* is an underserved, highly congested settlement of slum houses, posing major challenges to implement the public health control measures. The first four individuals who were detected to be infected with the SARS-CoV2 virus were identified to have 1093 close contacts, due to the highly congested living conditions. However, despite 2722 individuals living in 6,955 square meters (0.007 km^2^) and sharing toilets, infection was only detected by RT-qPCR in 85/1093 close contacts, although another 30 individuals were found to have had COVID-19 based on the presence of SARS-CoV-2 specific antibodies. Although the containment of infection in this underserved settlement is surprising, a larger slum area in Mumbai, which has a slightly lower population density (388,857.1/km^2^ in Bandaranayaka watta vs 277,136/km^2^ Dharvi-Mumbai), also successfully contained a COVID-19 outbreak [15]. Therefore, implementing the WHO’s pillars of the strategy of the public health response and aiming to contain the virus [16], does appear to be very effective in preventing outbreaks, even in areas, where it is most challenging to do so.

Although 18 (20.2%) of those who had COVID-19 were in the 61 to 80 years age group and 1 person over 80 years of age, no one developed moderate or severe COVID-19 illness. In fact, 86.5% individuals developed asymptomatic illness. Although 19% of infected individuals have shown to develop severe disease of which 5% become critically ill [17], none of these patients developed moderate or severe illness. 12.4% of this cohort had diabetes and 8/89 (9%) had cardiovascular disease, which are known risk factors for severe disease [18–20]. Therefore, environment or host factors that affect clinical disease severity should be further investigated.

In this study, to determine infection with the SARS-CoV-2 virus, we also assessed the SARS-CoV-2 specific total antibodies and Nabs. Nabs have shown to decay with time, especially in those with mild or asymptomatic infection [11]. Therefore, this shows while the total Abs persist for longer the Nabs appear to decay faster in mild and asymptomatic individuals. In order to understand the implications for long term immunity and vaccinations, it would be important to further understand that type of antibody responses that persist and if such antibodies could prevent re-infection. Indeed, one individual who was found to have an asymptomatic infection with SARS-CoV-2 in April was reinfected in December 2020. This person did not have any SARS-CoV-2 specific antibodies following infection. Therefore, careful epidemiological studies are necessary to determine the rates of re-infection and the type and quantity of antibodies that associate with protection as this information is crucial for vaccine development.

Due to the limited number of individuals who were positive by RT-qPCR (n = 89), we could only successfully carry out whole genomic sequencing in 2 viruses. One virus lineage corresponded to the European lineages associated with the large Italian outbreak (B.1) and an Iranian lineage (B.4) [21, 22]. This limited data, suggests that multiple SARS-CoV-2 strains were introduced to these communities from different sources and multiple strains circulated in this area. Of the two viruses, one carried the D614G mutation in the S protein, which have been associated with higher transmissibility and infectivity [4]. No mutations or deletions of other nucleotides associated with milder illness were detected, such as the SARS-CoV-2 variant with the deletion of 382 nucleotides in the ORF8 reading frame [23]. Since this study was carried out from April to May 2020, none of the current SARS-CoV2 variants that are associated with higher transmissibility that are currently emerging from many countries such as the N501Y or the E484K mutation were detected [24]. None of the other mutations in the RBD N439/R426, L452/K439, T470/N457, and Q498/Y484 that are associated with higher binding affinity of spike protein of the virus to the RBD were detected either [25]. Despite one of the viruses carrying the D614G mutation and despite this outbreak occurring in an extremely overcrowded area, it appears that only 89/2722 living in the 6,955 square meters (0.007 km^2^) area of the Bandaranayaka watta were infected.

There were several other limitations in this study. Although nasopharyngeal swabs were obtained from all the study participants, only a subset of the participants volunteered to give a blood sample for serological assays. Further some individuals were lost to follow up after 120–160 days. In few patients the sVNT assay for SARS-CoV-2 Nabs could not be measured due to inadequate serum volume.

In conclusion, we have described the transmission dynamics, serosurveillence data of a COVID-19 disease outbreak that occurred during the first wave in Sri Lanka in a highly crowded, lower socioeconomic area in Colombo, Sri Lanka. Since almost all infected individuals developed mild or asymptomatic illness and since the majority of those who were in this very closely packed housing area were not infected, it would be important to further study environmental and host factors that lead to disease severity and transmission.

## References

[1] Centre CR. Mortality analysis: John Hopkins University; 2020 [updated 21st December 202023rd December 2020]. Available from: https://coronavirus.jhu.edu/data/mortality.

[2] Organization WH. COVID-19 Virtual Press conference transcript—12 October 2020 2021. Available from: https://www.who.int/publications/m/item/covid-19-virtual-press-conference-transcript---12-october-2020.

[3] Fang Y, Nie Y, Penny M. Transmission dynamics of the COVID-19 outbreak and effectiveness of government interventions: A data-driven analysis. Journal of medical virology. 2020;92(6):645–59. doi: 10.1002/jmv.25750 ; PubMed Central PMCID: PMC7228381. 32141624 PMC7228381

[4] Korber B, Fischer WM, Gnanakaran S, Yoon H, Theiler J, Abfalterer W, et al. Tracking Changes in SARS-CoV-2 Spike: Evidence that D614G Increases Infectivity of the COVID-19 Virus. Cell. 2020. doi: 10.1016/j.cell.2020.06.043 ; PubMed Central PMCID: PMC7332439. 32697968 PMC7332439

[5] Control ECfDPa. Risk related to spread of new SARSCoV-2 variants of concern in the EU/EEA. Stockholm: 2020 29th December 2020. Report No.

[6] Epidemiology unit MoH, Sri Lanka. COVID-19, National Epidemiological Report, Sri Lanka Ministry of Health, Sri Lanka2020. Available from: https://www.epid.gov.lk/web/.

[7] Epidemiology unit MoH, Sri Lanka. Coronavirus disease 2019 (COVID-19)—Situation Report– 31.08.2020. Epidemiology Uni, 2020 31.08.2020. Report No.

[8] WHO. World Health Organization. Coronavirus disease (COVID-19) technical guidance: early investigations. World Health Organization; 2020.

[9] WHO. Clinical management of severe acute respiratory infection when novel coronavirus (2019-nCoV) infection is suspected: interim guidance. WHO, 2020 28th January 2020. Report No.

[10] Tan CW, Chia WN, Qin X, Liu P, Chen MI, Tiu C, et al. A SARS-CoV-2 surrogate virus neutralization test based on antibody-mediated blockage of ACE2-spike protein-protein interaction. Nature biotechnology. 2020;38(9):1073–8. doi: 10.1038/s41587-020-0631-z . 32704169

[11] Chandima Jeewandara DJ, Laksiri Gomes, Ananda Wijewickrama, Eranga Narangoda, Damayanthi Idampitiya, Dinuka Guruge, et al. SARS-CoV-2 neutralizing antibodies in patients with varying severity of acute COVID-19 illness. Research Square2020. 10.1038/s41598-021-81629-2 PMC7819970 33479465

[12] Phylogenetic Assignment of Named Global Outbreak LINeages [Internet]. 2020 [cited 16.09.2020]. Available from: https://github.com/cov-lineages/pangolin.

[13] Singer JG, R.; Cotten, M.; Robertson, D. CoV-GLUE: A Web Application for Tracking SARS-CoV-2 Genomic Variation2020. Available from: https://www.preprints.org/manuscript/202006.0225/v1.

[14] Newcombe RG. Confidence Intervals for Proportions and Related Measures of Effect Size. First ed: CRC Press; 2013. 470 p.

[15] Golechha M. COVID-19 Containment in Asia’s Largest Urban Slum Dharavi-Mumbai, India: Lessons for Policymakers Globally. J Urban Health. 2020;97(6):796–801. doi: 10.1007/s11524-020-00474-2 ; PubMed Central PMCID: PMC7437383. 32815097 PMC7437383

[16] WHO. A new Strategic Preparedness and Response Plan was published on 24 February 2021. WHO Headquarters (HQ), WHO Worldwide: World Health Organization; 2021.

[17] Wu Z, McGoogan JM. Characteristics of and Important Lessons From the Coronavirus Disease 2019 (COVID-19) Outbreak in China: Summary of a Report of 72314 Cases From the Chinese Center for Disease Control and Prevention. JAMA: the journal of the American Medical Association. 2020. doi: 10.1001/jama.2020.2648 . 32091533

[18] Wang G, Wu C, Zhang Q, Yu B, Lu J, Zhang S, et al. Clinical characteristics and the risk factors for severe events of elderly coronavirus disease 2019 patients. Zhong Nan Da Xue Xue Bao Yi Xue Ban. 2020;45(5):542–8. doi: 10.11817/j.issn.1672-7347.2020.200292 . 32879104

[19] Albitar O, Ballouze R, Ooi JP, Sheikh Ghadzi SM. Risk factors for mortality among COVID-19 patients. Diabetes research and clinical practice. 2020;166:108293. doi: 10.1016/j.diabres.2020.108293 ; PubMed Central PMCID: PMC7332436. 32623035 PMC7332436

[20] Tian W, Jiang W, Yao J, Nicholson CJ, Li RH, Sigurslid HH, et al. Predictors of mortality in hospitalized COVID-19 patients: A systematic review and meta-analysis. Journal of medical virology. 2020. doi: 10.1002/jmv.26050 . 32441789 PMC7280666

[21] Pangolin. SARS-CoV2 lineages 2020. Lineages assigned by the software tool pangolin ]. Available from: https://cov-lineages.org/descriptions.html.

[22] Rambaut A, Holmes EC, O’Toole A, Hill V, McCrone JT, Ruis C, et al. A dynamic nomenclature proposal for SARS-CoV-2 lineages to assist genomic epidemiology. Nat Microbiol. 2020. doi: 10.1038/s41564-020-0770-5 . 32669681 PMC7610519

[23] Young BE, Fong SW, Chan YH, Mak TM, Ang LW, Anderson DE, et al. Effects of a major deletion in the SARS-CoV-2 genome on the severity of infection and the inflammatory response: an observational cohort study. Lancet. 2020;396(10251):603–11. doi: 10.1016/S0140-6736(20)31757-8 ; PubMed Central PMCID: PMC7434477. 32822564 PMC7434477

[24] SARS-CoV-2 Variants [Internet]. World Health Organization; 2020. Emergencies preparedness, response; 31st December 2020. Available from: https://www.who.int/csr/don/31-december-2020-sars-cov2-variants/en/

[25] Yi C, Sun X, Ye J, Ding L, Liu M, Yang Z, et al. Key residues of the receptor binding motif in the spike protein of SARS-CoV-2 that interact with ACE2 and neutralizing antibodies. Cellular & molecular immunology. 2020;17(6):621–30. doi: 10.1038/s41423-020-0458-z ; PubMed Central PMCID: PMC7227451. 32415260 PMC7227451

